# Comparison of acute skin reaction following morning versus late afternoon radiotherapy in patients with breast cancer who have undergone curative surgical resection

**DOI:** 10.1093/jrr/rrt141

**Published:** 2014-01-01

**Authors:** Jae Myoung Noh, Doo Ho Choi, Hyojung Park, Seung Jae Huh, Won Park, Seung Won Seol, Bae Kwon Jeong, Seok Jin Nam, Jeong Eon Lee, Won-Ho Kil

**Affiliations:** 1 Department of Radiation Oncology, Samsung Medical Center, Sungkyunkwan University School of Medicine, #50 Irwon-dong, Gangnam-gu, Seoul 135-710, Republic of Korea; 2 Department of Radiation Oncology, Gyeongsang National University Hospital, Chiram-dong, Jinju, Gyeongsangnam-do 660-702, Republic of Korea; 3 Department of Surgery, Samsung Medical Center, Sungkyunkwan University School of Medicine, #50 Irwon-dong, Gangnam-gu, Seoul 135-710, Republic of Korea

**Keywords:** circadian rhythm, radiotherapy, radiation-induced dermatitis, breast cancer

## Abstract

We investigated the relationship between the time of radiotherapy (RT) and treatment outcomes in breast cancer. Patients with pathologic T1–2N0–1 breast cancer who received adjuvant RT in the morning (before 10:00 AM) or late afternoon (after 3:00 PM) were eligible for inclusion in this study. We retrospectively compared the clinicopathologic characteristics, acute skin reaction, and survival outcomes according to the time of RT. The median follow-up duration was 83 months (range, 10–131 months). From the 395 eligible patients, 190 (48.1%) and 205 (51.9%) patients were classified into the morning RT group and the afternoon RT group, respectively. The clinicopathologic characteristics were relatively well balanced between the treatment groups, except for pathologic N-stage (*P* = 0.0409). Grade 2 or higher acute skin reaction according to the Radiation Therapy Oncology Group criteria was observed in 39 (9.9%) patients, with a higher frequency in the afternoon RT group than the morning RT group (13.7% vs 5.8%, respectively; *P* = 0.0088). There was no difference in the failure patterns or survival outcomes between the treatment groups. RT in late afternoon was associated with increased Grade 2 or more skin reaction after RT for breast cancer patients, but treatment outcomes did not differ according to the time of RT. Individualized considerations for treatment should be taken into account to reduce the risk of skin reactions.

## INTRODUCTION

Normal cell physiology includes a circadian rhythm that is coordinated by the suprachiasmatic nuclei of the anterior hypothalamus [1–3]. Several clock genes are involved in the molecular feedback loops that control circadian timing [3, 4]. Circadian cycles are also involved in cell cycle regulation and tumor suppression by clock-controlled genes such as the *MYC*, *cyclin-D1* and *WEE1* genes [5, 6]. Consequently, tolerance and response to anticancer treatment are modulated by circadian changes [7, 8]. Given this background, therapeutic approaches using circadian rhythm, so-called chronotherapy, have been widely investigated. The improved anticancer efficacy of chemotherapeutic agents has been observed in experimental models of chronotherapy [9, 10]. Clinical trials of chronotherapy have also demonstrated less toxic and more effective results in patients with metastatic breast or colorectal cancer [11–13].

According to the circadian variation in the cell cycles in human oral or rectal mucosa [14, 15], response to ionizing radiation also has circadian variations [16, 17]. Several randomized trials have investigated the relationship between the time of radiotherapy (RT) and incidence of oral or rectal mucositis [18–20]. The time of irradiation was associated with the incidence of mucositis, lack of tumor response or locoregional control [18–20]. These studies included patients with head and neck cancer or uterine cervical cancer. Other primary cancers have rarely been investigated with regard to the time of RT. Our institution has day and night shifts because of a heavy workload. Therefore, some of our patients receive RT in morning and others in the late afternoon. In this study, we investigated the relationship between the time of RT and treatment outcomes in patients with breast cancer who underwent curative surgical resection.

## MATERIALS AND METHODS

Between October 2001 and December 2006, a total of 1956 patients underwent adjuvant whole breast or chest wall RT for breast cancer at Samsung Medical Center. From these patients, we selected 682 patients who received 80% or more of their overall fractions in the morning (between 7:00 AM and 10:00 AM) or in the late afternoon (between 3:00 PM and 10:00 PM). Then, we excluded patients who (i) underwent surgical resection at an outside hospital and were referred back to the hospital after RT (*n* = 126), (ii) who received neoadjuvant chemotherapy (*n* = 28), or (iii) had recurrent breast cancer (*n* = 11). We retrospectively reviewed the medical and pathologic records of 400 patients with pathologic T1–2N0–1 stage. Five patients without available estrogen receptor (ER), progesterone receptor (PR), and human epidermal growth factor receptor-2 (HER-2) receptor status were also excluded, which left 395 eligible patients. This study was approved by the Institutional Review Board of Samsung Medical Center (2013-07-015).

Nuclear grade and status of immunohistochemical (IHC) staining for ER, PR and HER-2 were recorded on pathologic review. Allred scores ranging from 3–8 were defined as having positive immunoreactivity for ER and PR. Positive HER-2 was determined using IHC 3+ staining or amplification on fluorescence *in situ* hybridization. According to the receptor status, patients were classified into three groups: luminal (ER- or PR-positive), triple-negative (ER-, PR-, and HER-2-negative), or HER-2 overexpressing (ER- and PR-negative, and HER-2 positive).

Through 2002, radiation was delivered to the whole breast or chest wall at a total dose of 50.4 Gy in 28 fractions (*n* = 67). Since 2003, the delivered dose has been 50 Gy in 25 fractions (*n* = 328). A tangential technique with 4- or 6-MV photon beams generated by linear accelerator (Varian Medical System, Palo Alto, CA, USA) was used for whole breast or chest wall irradiation. The field borders of tangential fields were as follows: superior, the head of the clavicle, which could be extended according to palpable breast tissue; inferior, 1.5–2 cm below the inframammary fold; medial, midline; lateral, usually midaxillary line, which also could be modified according to palpable breast tissue. By using computed tomography (CT)-based treatment planning, hotspots were limited to 110% of the prescribed dose and preferably not 107%. Dose calculation was carried out by fast photon calculation. The primary tumor bed was boosted in 367 (92.9%) patients. The boost dose ranged from 9–12 Gy with a daily dose of 2.0–3.5 Gy according to resection margin status. An electron beam was used for primary tumor bed boost. Nine (2.3%) patients received irradiation of the supraclavicular fossa. Adjuvant chemotherapy was delivered to 303 (76.7%) patients, and hormonal therapy was an additional treatment for 253 (64.1%) patients (according to hormonal receptor status).

The median follow-up duration, calculated from the date of surgery, was 83 months (range, 10–131 months). Acute skin reactions were assessed without clinicopathologic information by two radiation oncologists according to the Radiation Therapy Oncology Group (RTOG) criteria. The chi-square test was used to examine the differences in clinicopathologic characteristics between the treatment groups. Overall survival (OS) was defined as the time from the date of surgery to death from any cause, and disease-free survival (DFS) was defined as the time from the date of surgery to breast cancer relapse or death. OS and DFS were estimated using the Kaplan–Meier test and compared by log-rank test. Cox proportional hazards regression analysis was used for multivariate analysis. SAS version 9.1.3 software (SAS Institute Inc., Cary, NC, USA) was used for statistical analysis. A *P*-value < 0.05 was considered statistically significant.

## RESULTS

The median age of the 395 eligible patients was 47 years (range, 22–81 years). Among the patients, 190 (48.1%) and 205 (51.9%) patients were classified into the morning RT group and the afternoon RT group, respectively. Among the afternoon RT group, 32 (15.6%) patients received 80% or more of overall fractions after 6:00 PM. Invasive ductal carcinoma accounted for 85.0% of all tumors. The characteristics of the patients in each group are presented in Table 1. Pathologic N-stage was different between the treatment groups (*P* = 0.0409), but other characteristics were generally well balanced between the treatment groups.

**Table 1.** Patients characteristics by treatment group

Characteristics		Morning (*n* = 190)	Afternoon (*n* = 205)	*P*
Median age (years, range))		47 (25–81)	46 (22–77)	
Location	Right	101 (53.2%)	99 (48.3%)	0.6221
	Left	88 (46.3%)	105 (51.2%)	
	Bilateral	1 (0.5%)	1 (0.5%)	
Surgery	Breast-conserving	185 (97.4%)	199 (97.1%)	0.8586
	Mastectomy	5 (2.6%)	6 (2.9%)	
pT-stage*	1	137 (72.1%)	161 (78.5%)	0.1379
	2	53 (27.9%)	44 (21.5%)	
pN-stage*	0	129 (67.9%)	158 (77.1%)	0.0409
	1	61 (32.1%)	47 (22.9%)	
Nuclear grade	Low	30 (15.8%)	42 (20.5%)	0.1308
	Intermediate	81 (42.6%)	99 (48.3%)	
	High	71 (37.4%)	59 (28.8)	
	Unknown	8 (4.2%)	5 (2.4%)	
ER	Positive	119 (62.6%)	140 (68.3%)	0.2368
	Negative	71 (37.4%)	65 (31.7%)	
PR	Positive	107 (56.3%)	128 (62.4%)	0.2155
	Negative	83 (43.7%)	77 (37.6%)	
Molecular subtype	Luminal	125 (65.8%)	145 (70.7%)	0.4335
	Triple-negative	46 (24.2%)	46 (22.4%)	
	HER-2 overexpressing	19 (10.0%)	14 (6.8%)	

*Pathologic staging according to the American Joint Committee on Cancer 7th edition, 2010. ER = estrogen receptor, PR = progesterone receptor, HER-2 = human epidermal growth factor receptor-2.

RTOG Grade 2 or higher acute skin reaction developed in 39 (9.9%) patients, with a higher frequency in the afternoon RT group (28 patients, 13.7%) than in the morning RT group (11 patients, 5.8%, *P* = 0.0088). During the follow-up period, 33 (8.4%) patients experienced breast cancer recurrence. At the first site of recurrence, the overall incidence of locoregional recurrence and distant metastasis was not different between the two groups (Table 2). The 7-year DFS and OS rates of all patients were 93.4% and 96.3%, respectively. The 7-year DFS rate was 92.7% in the morning RT group and 94.0% in the afternoon RT group, respectively (*P* = 0.9742). OS was also similar between the groups (7-year rate, 96.0% vs 95.9%, respectively, *P* = 0.8485). Decreased DFS was observed in patients with triple-negative or HER-2 overexpressing subtypes (*P* = 0.0168) and higher nuclear grade (*P* = 0.0487, Table 3). OS also decreased in patients with triple-negative or HER-2 overexpressing subtypes, but this was only marginally significant (*P* = 0.0597). On multivariate analysis, there was no significant prognostic factor affecting DFS or OS (Table 4), while molecular subtype was a marginally significant factor affecting DFS (hazard ratio, 1.693; *P* = 0.0565).

**Table 2.** Patterns of first recurrence according to treatment group

Treatment	Locoregional recur		Distant metastasis		Contralateral	
	No. (%)	*P*	No. (%)	*P*	No. (%)	*P*
Morning	6 (3.2%)	0.8936	7 (3.7%)	0.6733	3 (1.6%)	0.7254
Afternoon	6 (2.9%)		6 (2.9%)		5 (2.4%)

**Table 3.** Prognostic factors affecting disease-free survival and overall survival on univariate analysis

Variables	Disease-free survival		Overall survival	
	7 year rate	*P*	7 year rate	*P*
Age		0.4513		0.4105
<40 years	93.5%		95.1%	
≥40 years	93.3%		96.1%	
Treatment group		0.9742		0.8485
Morning	92.7%		96.0%	
Afternoon	94.0%		95.9%	
pT-Stage*		0.9533		0.3763
1	93.6%		96.4%	
2	92.6%		94.4%	
pN-Stage*		0.5621		0.9846
0	94.6%		96.2%	
1	90.2%		95.2%	
Molecular subtype		0.0168		0.0597
Luminal	96.2%		97.6%	
Triple-negative	88.2%		92.0%	
HER-2 overexpressing	84.1%		93.3%	
Nuclear grade		0.0487		0.1615
Low	100%		97.9%	
Intermediate	94.1%		97.1%	
High	89.6%		93.4%	

*Pathologic staging according to the American Joint Committee on Cancer 7th edition, 2010. HER-2 = human epidermal growth factor receptor-2.

**Table 4.** Prognostic factors affecting survival outcomes on multivariate analysis

Factors	Disease-free survival		Overall survival	
	*P*	Hazard ratio (95% C.I.)	*P*	Hazard ratio (95% C.I.)
Treatment group	0.8469	1.073 (0.526–2.188)	0.7714	0.867 (0.330–2.275)
pT-Stage*	0.3914	0.682 (0.287–1.631)	0.5814	1.336 (0.477–3.742)
pN-Stage*	0.4631	1.328 (0.622–2.837)	0.8719	0.916 (0.315–2.661)
Nuclear grade	0.1538	1.577 (0.843–2.950)	0.2327	1.674 (0.718–3.903)
Molecular subtype	0.0565	1.693 (0.986–2.907)	0.3482	1.409 (0.688–2.882)

*Pathologic staging according to the American Joint Committee on Cancer 7th edition, 2010.

## DISCUSSION

In this study, the time of RT was associated with increased Grade 2 or higher acute skin reaction. About 10% of all patients developed significant acute skin toxicity, which was lower than the previously reported incidence [21, 22]. Avoidance of hotspots receiving excessive dose by CT-based planning and the relatively small breast size of the Korean population compared with the Western population could be a possible cause for this difference. Although this retrospective study did not include other possible factors affecting skin reaction, a chronological difference in skin reaction after breast or chest wall RT was observed. To the best of our knowledge, the present study is the first study demonstrating a chronological effect of RT on breast cancer. Given the day and night shifts, 15.6% of the afternoon RT group received RT later than 6:00 PM, but we did not differentiate the late evening treatment from the late afternoon treatment.

Genetic polymorphisms in DNA repair genes and accompanying G2 phase prolongation have been known to be associated with an acute response to RT [23, 24]. Increased radiosensitivity has been observed in *BRCA* mutation carriers [25], while the incidence of Grade ≥2 acute skin toxicity has not been associated with the *BRCA* mutation in patients with breast cancer who have undergone breast-conserving surgery followed by RT [26]. In addition to these factors, circadian rhythm in the cell cycle could also influence acute reactions [14, 15]. Based on observations that the most radiosensitive phase of the cell cycle (G2-M) occurs in the late afternoon, two randomized studies have evaluated acute oral mucositis in patients who have received head and neck RT [19, 20]. Grade 3 or greater oral mucositis was more prevalent in the evening RT group than in the morning RT group, although this was not statistically significant. Treatment outcomes such as response rate or locoregional control were not different between the treatment groups. Similar results were found in the present study. Using similar cutoffs for classifying the treatment groups, increased significant skin reaction was observed without a difference in treatment outcomes. Another randomized study examined radiation-induced intestinal mucositis in patients with cervical cancer [18]. In contrast to oral mucositis, radiation-induced bowel toxicities were higher in the morning RT group. Although different findings were observed, the common suggestion from these studies was that the potential contribution of the circadian rhythm in the cell cycle of tumor or normal tissue should be taken into account [18–20].

Breast cancer is one of the most common malignancies in women [27]. A large proportion of patients receive adjuvant whole breast or chest wall RT after surgical resection according to the risk of locoregional recurrence [28–30]. About one-third of these women experience acute skin reaction following RT [21, 22], and given that this is associated with reduced quality of life, several trials have been performed in an attempt to minimize skin reactions [21, 22, 31]. A trial using intensity-modulated radiation therapy showed significantly reduced occurrence of moist desquamation [22]. In addition to reduction of skin dose, modification of treatment time might be another alternative for patients who have large-sized breasts. Clinical investigations regarding the chronological effect of RT for other malignancies are now needed. For example, colorectal cancer could be a good candidate for chronotherapy, reported to be effective in patients with metastatic disease [11, 13].

## CONCLUSION

In summary, the time RT was given was associated with the occurrence of skin reactions in breast cancer patients. Although there were some limitations for this study due to its retrospective nature, we found that the clinicopathologic characteristics were relatively well balanced between the treatment groups. Most importantly, the treatment outcomes were not different. In addition to clinical, genetic and dosimetric factors affecting RT-related skin toxicity, the chronological aspect of RT should also be taken into account in treating patients with breast cancer.
